# A New Paradigm for Threat Agnostic Biodetection: Biological Intelligence (BIOINT)

**DOI:** 10.1089/hs.2023.0072

**Published:** 2024-02-19

**Authors:** Thomas Knight, Swati Sureka

**Affiliations:** Thomas Knight, PhD, is Co-Founder and Ginkgo Fellow, Ginkgo Bioworks, Boston, MA.; Swati Sureka, MSc (Oxon, Edin), is Business Operations Manager; Ginkgo Bioworks, Boston, MA.

**Keywords:** Biological intelligence, Emerging threats, Early warning, Microbial ecology, Metagenomic sequencing

## Introduction

At the dawn of the Cold War, the US Air Force tapped a group of scientists—known as the Beacon Hill study group—to explore new approaches to understanding what was happening behind the Iron Curtain.^1^ Dr. Edwin Land, the chief executive officer of Polaroid, recognized that aerial photography was potentially “the most powerful single tool for acquiring information” and represented “a unique opportunity for comprehensive intelligence.”^2^ This assessment paved the way for U-2 reconnaissance aircraft and, later, the CORONA satellites and follow-on satellite imaging systems. But Land foresaw even greater potential. He emphasized that interpretation of aerial photographs provided nearly unlimited military, civilian, and peacetime uses, and he was right: geospatial intelligence (GEOINT) based on remote imaging (and remote sensing broadly) became, and remains, a pillar of security, economy, and human and environmental wellbeing.

Today, mapping and understanding the *biological* world presents an opportunity of similar significance. Like imaging technology, sequencing and analysis of nucleic acids in environmental samples provide a massive and rich trove of information, in an accessible and versatile form. We have barely begun to leverage its potential. A paradigm shift is needed—one of consistently collecting, organizing, and utilizing our global environment's biological information as we currently do for other information sources. The persistent and pervasive sampling and metagenomic analysis of environmental DNA and RNA forms the basis of this new paradigm, which we call biological intelligence (BIOINT).

BIOINT builds upon and draws inspiration from a mosaic of existing biodetection systems and proposals, recognizing that these initiatives represent necessary but insufficient scope. Many existing approaches to infectious disease surveillance are largely reactive: a pattern of clinical cases with similar symptomatic profiles emerges, triggering efforts to identify, characterize, and respond to the underlying threat and monitor its progression. This pattern played out during the COVID-19 pandemic—viral surveillance and tracking efforts were spun up during its peak but have now wound down worldwide. Persistent environmental surveillance, conducted both during and in between crises, provides greater opportunity to achieve early warning and richer data on broader microbial and macroorganism ecology rather than any single threat, and has certainly begun to play a role in infectious disease surveillance.

The potential of analyzing environmental genomes has long been recognized for public health and biodefense, as well as biodiversity conservation, ecosystem health, research, and other purposes. Existing initiatives vary widely in their scope—from large-scale metabarcoding approaches for taxonomic, but not functional, characterization,^3,4^ to the democratization of genomic and metagenomic analysis for localized management of ecosystem health.^5^ A recent proposal for large-scale environmental metagenomic analysis strives for the creation of national genomic repositories—a laudable but incomplete goal.^6^

BIOINT goes further, encompassing collection and metagenomic analysis of environmental samples that are drawn from a set of sampling sites that are identified (and updated) based on risk modeling (such as ecological niche modeling^7^), state-of-the-art analytics to rapidly characterize threats and model potential interventions and response scenarios, integration with other sources of data^8^ to produce point-of-decision intelligence, and a global, rather than fragmented or federated, approach to data aggregation). The latter is necessary to glean valuable insights given the interconnectedness of human populations and biological systems and the dynamism and rapid pace of change within the biological world.

## The Promise of BIOINT

National and global security, economic competitiveness, public health, and environmental protection are entangled. In biology, as in other domains, threats are emerging at an accelerating pace and with ever-increasing stakes, driven by climate change, habitat disruption, and global interconnectedness. Broadening access to biotechnology is exacerbating concerns over its accidental or intentional misuse. While the COVID-19 pandemic elevated health security on the global stage, there are already signs it is being deprioritized amid the challenges of armed conflict and other shifting geopolitical conditions^9^—when in fact, these circumstances should redouble global focus and attention on BIOINT.

The biodefense and public health value of BIOINT far exceeds that of only collecting “raw” environmental sequences from various sites, in several key ways:

- **Persistence:** Continuous collection establishes environmental baselines at each site through time, enabling the rapid detection of anomalies. The always-on approach generates valuable insights both during crises, when attention to a specific threat ramps up while others are neglected, and in interpandemic periods, when environmental genomic data of any kind is woefully scarce. This approach is akin to satellite imaging networks maintaining “continuous custody” of targets of interest, with environmental baselines directing targeted collection when indicated.
- **Pervasiveness:** A distributed geographical network enables baselining of threat distribution and its localization across time and space. Strategic selection of heterogeneous nodes where biological threats are likely to emerge allows for the efficient identification of threats from multiple domains and sources of risk. Collection may not be possible everywhere due to geopolitical or physical barriers (eg, in states that withhold data), but the interconnectedness of human–environmental systems still allows biointelligence insights into inaccessible locales (eg, from waterways that carry biological material across national borders).
- **Agnostic approach:** Infectious disease surveillance, biodiversity conservation efforts, and other existing large-scale environmental biomonitoring systems generally filter their analyses through amplification or enrichment of a particular subset of signals of interest. While this is an effective and efficient approach in many ways, it can also filter out novel signals from previously unknown specimens. BIOINT, in the long-term, will take a metagenomics-first approach to enable true agnostic biodetection.
- **Depth of analytic insight:** State-of-the-art computational tools leveraging artificial intelligence and machine learning are or will be able to identify and characterize threats, ascertain whether and how they were manipulated, provide insights as to their origins, and predict their trajectories under different response scenarios with increasing fidelity.
- **Integrated intelligence:** Insights from these continuous environmental baselines are layered with other forms of data, such as proteomics; public health data on health system status, syndromic surveillance, and surge testing; geospatial (including environmental and meteorological) data; ecological data on hosts, vectors, and dynamic conditions; media and social media data indicating population sentiment and behavior; and travel and trade data to create a powerful form of intelligence (Figure).

**Figure.**
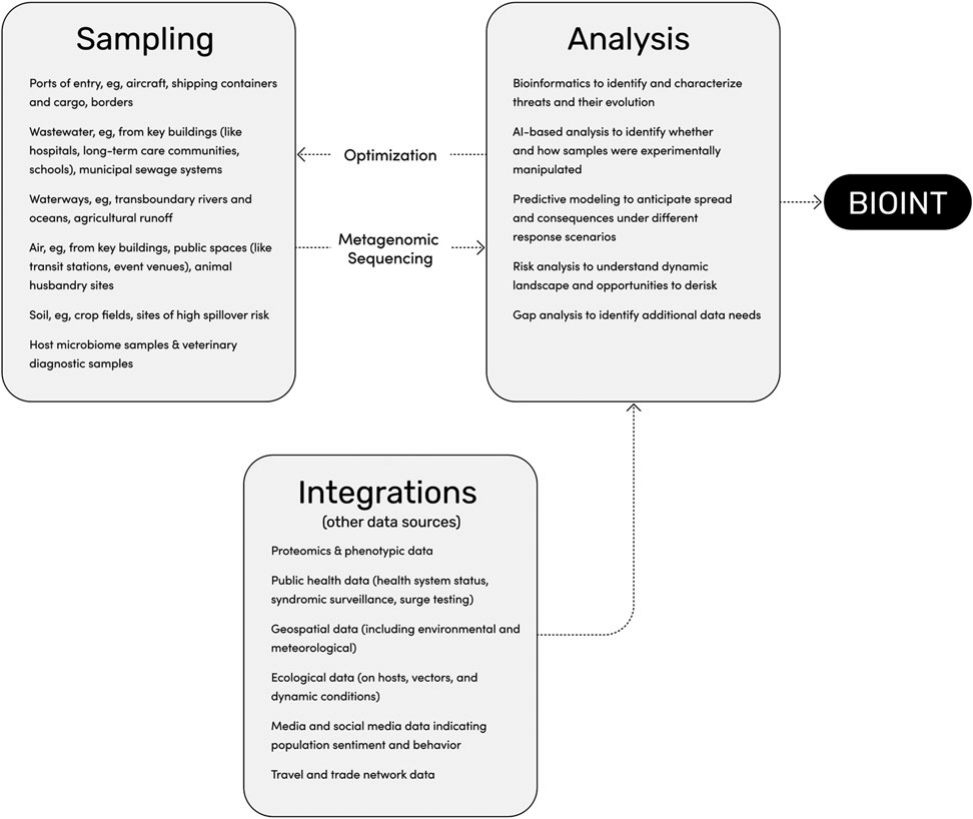
Overview of BIOINT generation process for health security applications. Creating BIOINT requires several core components: a risk-informed environmental sampling strategy, a metagenomic sequencing-based approach to produce threat-agnostic genomic data, integration of a variety of other types of data and intelligence, and a robust analytic toolkit to generate point-of-decision intelligence (and further optimize data collection practices).

Additionally, BIOINT must be inherently global—not only in scale of collection but also in aggregation of insights. The contours of intelligence look different in the 21st century than in the Cold War. Rampant misinformation and disinformation campaigns have unprecedented reach through the internet; nonstate actors increasingly collect, interpret, and act upon data with intelligence value; and significant insights are increasingly found within open-source information.^10^ Against this backdrop, intelligence, including BIOINT, is inherently multilateral—and its efficacy increasingly relies upon rapidly validating, contextualizing, and strategically sharing (rather than concealing) it across disciplines and national borders.

While the focus of this commentary is on the health security applications of BIOINT to achieve early detection, localization, characterization, and attribution of natural and engineered biological threats and facilitate timely, appropriate responses with point-of-decision intelligence, the potential for BIOINT infrastructure as envisioned herein is also vast and diverse. BIOINT can support environmental protection, food security (of crops and farm animals), and conservation efforts. As an illustrative example, a recent initiative to layer genetic analysis with physical evidence, shipping data, and financial and communication records revealed unprecedented insights into and facilitated action on networks of illegal ivory trade formed by transnational criminal organizations.^11^ The NSF National Ecological Observation Network (NEON) program, collecting and sequencing historical environmental microbial samples from water, air, and soil at 81 carefully selected sites across the US, is an early example of what part of a comprehensive environmental collection plan might look like at scale.^12^

As the global bioeconomy grows, BIOINT can be leveraged to secure flows of genetic resources while preserving national and community sovereignty alongside commercial intellectual property rights and unleash new opportunities for innovation and economic growth. Increasingly, the availability of biological information, especially large databases of metadata-tagged nucleic acid sequences, is a core intellectual asset. It forms the basis for engineering novel biological systems, including the data necessary to train machine learning systems. Recognizing this, the White House recently announced a “bold goal” of sequencing the genomes of 1 million microbial species and characterizing newly discovered genes to further cross-cutting advances in biotechnology and biomanufacturing.^13^ Engineered biology is poised to have transformational impacts across the economy,^14^ from agriculture to consumer goods to energy, as well as healthcare and health security.^15^

## A Roadmap for BIOINT

We believe the scaled implementation of a robust BIOINT system is possible on the timescale of roughly a decade. The technology needed to deploy BIOINT largely already exists and is rapidly improving. While resource constraints remain, these are becoming ever more surmountable. BIOINT's successful implementation requires creative and robust approaches to facilitating data sharing and operationalization of intelligence into action. This section explores each of these dimensions.

### Technology

Metagenomic sequencing of environmental samples is at the core of BIOINT, and this technology is full of promise. While PCR amplification or other forms of barcode-based targeting generally narrow the aperture of genomic analysis today, we envision a transition to an agnostic, metagenomics-first approach in time, with targeted amplification and enrichment used in follow-up analyses to enhance analytic depth.

The technology for multithreat biodetection in environmental samples is already quite advanced. Researchers have demonstrated the efficacy of wastewater-based detection for many human pathogens, along with genetic markers of antimicrobial resistance.^16,17^ Other environmental samples—such as those from soil, air, water, and animal host and vector populations and microbiomes—increasingly can be layered on top of wastewater collection to gain a richer picture of the biological environment and push collection closer to the sites of disease emergence. Innovation in sampling (along with processing and analysis pipelines) is driving progress on this front, leveraging automation, ruggedized tools, and xenosurveillance techniques^18^ to improve insights into environmental reservoirs.

Transitioning to a metagenomics-first, truly threat-agnostic approach is critical, as amplification- and enrichment-based approaches may not be effective at detecting previously unknown threats. While substantial investment and technical development are needed to realize this transition, several converging trends in the coming decade are encouraging. Automation enables cheaper and faster sequencing, while achieving greater depth and longer and more accurate reads. Combining long- and short-read sequencing allows the resolution of complex metagenomic samples.^19^ Technologies to selectively deplete abundant background molecules from environmental samples make it easier to detect low-abundance organisms. Next-generation techniques like Linked-Reads, genomic crosslinking (Hi-C), and single-cell encapsulation increasingly associate mobile and extrachromosomal genes with organisms. Basic sequencing technology is also improving; for example, innovation in lyophilized reagents is expected to reduce reliance on the cold chain, with striking implications for generating BIOINT in remote or low-resource settings.

Bioinformatic techniques are improving our ability to process and analyze this wealth of sequencing data. Cloud-based platforms allow for greater standardization and interoperability along with custom analysis pipelines.^20^ High-quality genomic reference databases continue to grow and improve our ability to rapidly identify concerning mutations, variants, and pathogens. New algorithms, including machine learning and artificial intelligence approaches, reduce reliance on these databases, allowing us to predict function. Progress in computational technologies is further improving our ability to draw epidemiological inferences, predict the impacts of different response scenarios, and meaningfully integrate bioinformatic insights with other data layers.

### Resources

BIOINT has the potential to become a critical national and global security asset and global public good whose value extends far beyond public health applications. While some aspects of BIOINT collection infrastructure may build upon and draw resources from health systems (eg, through infectious disease surveillance programs), the broader use cases and persistent strategic and intelligence value of BIOINT cannot be realized if it is subject to the panic-and-neglect cycles of public health funding. BIOINT's implementation and financing should therefore be driven primarily with the resources of national security agencies across like-minded countries. As the bioeconomy develops, demand from the private sector will spur additional funding for BIOINT, similar to the development of the geospatial intelligence data market.

Insofar as BIOINT offers the opportunity to intervene in biological events before they can cause mass damage, it has the potential to be a highly cost-effective approach.^21^ The technologies underlying BIOINT, such as agnostic sequencing, can be costly, and resource constraints are often cited as a major hurdle to more pervasive environmental sampling and analysis. This is a reasonable concern, but the actual impacts of resource limitations in the intermediate term often are overstated.

The aforementioned technological advancements are rapidly driving down BIOINT's costliness. Autosampling technologies reduce the manual burden of collection. The costs of large-scale sequencing are falling faster than in almost any other technology area. The National Human Genome Research Institute has analyzed the declining costs of sequencing per raw megabase and per human genome over the period 2001-2021, finding that both have very substantially outpaced Moore's law (the computer industry trend describing the doubling of computational power every 2 years) since the adoption of next-generation sequencing technologies in 2008.^22^ Progress in high-performance computing is further improving the capacity and cost-effectiveness of complex data analysis. Projecting that these trends will continue, even marginally, the cost constraints that are front-of-mind for leaders and policymakers will soon become obsolete, enabling a far greater scale and scope of BIOINT generation.

In the meantime, analytical tools can optimize the allocation of scarce resources, ensuring that sampling focuses on geographic locations that provide the highest analytic utility: this could include sites with the highest risk of spillover for known zoonotic threats or greatest visibility into microbial flows within air transit networks. A risk-informed approach to sampling can help maximize the value of BIOINT generated within stringent resource constraints. Risk-informed surveillance sampling logics can be applied across endemic and epidemic risks, and across both natural and engineered threats, to achieve efficient breadth and depth of coverage without the cost of exhaustive sampling of all potential sites of interest. Resource-efficient deployment of BIOINT infrastructure is further aided by a virtuous cycle whereby BIOINT is fed back into probabilistic models of threat emergence and spread, which can inform site selection, allowing for an agile BIOINT network that is responsive to changing risk conditions. Importantly, these sites have strategic value not only on the global scale for security purposes but also on the local and national scale for a wide variety of other applications, increasing the likelihood of buy-in at multiple levels.

Several examples illustrate how sampling can be designed for resource-efficient coverage:

- Sampling at major transit hubs and ports of entry provides early warning for broad patterns of global spread,^23,24^ and the selection of nodes and sampling strategies at each node can be optimized for geographic and risk coverage using data on travel and trade networks. Such a network is already being stood up today.
- Ecological niche mapping uses a variety of ecogeographic and demographic variables to model probabilistic geographical distributions of zoonotic spillover^25^—areas with ostensibly suitable environmental conditions for emergence that have few reported instances indicate sites with problematic gaps in data. Threat agnostic approaches to site selection are tied to broad-based risk factors—for instance, sites of deforestation or other economic activity linked with habitat disruption are more likely to display novel, risky patterns of interaction between humans and animal hosts or vectors.^26,27^ A recent study leveraging metagenomic sequencing to track antibiotic resistance genes across more than 100 countries was able to reveal regional patterns of transmission and horizontal transfer, thereby identifying geographic targets for BIOINT collection.^17^
- Beyond spillover events, persistent analysis of the environment surrounding pathogen research laboratories, particularly in densely populated areas, can provide early warning to breaches of biocontainment.^28^
- Sites can be chosen to provide insights into jurisdictions that might withhold or obfuscate biological threat data, such as through borders or connecting waterways.

### Data Sharing

Some types of intelligence are, by nature, secret; their disclosure is limited to protect means and methods of collection and analysis. BIOINT is distinctive, in that many of its core capabilities are enabled by commercially derived and available technologies. Moreover, many of BIOINT's applications and use cases, particularly in health security, are *only* achievable through timely data exchange. A federated system, in which intelligence is collected and used on local and national scales without being aggregated to track global scale trends, holds only a small fraction of the value that can be derived from meaningful global aggregation, given the rapid global mobility of biological threats. BIOINT has a broad remit, and multinational and multisectoral cooperation is needed to deliver on the anticipated benefits to bioeconomic development alongside health security goals.

However, biological data—in particular, pathogen specimens and genomic sequences—have a complex legacy that needs to be considered for BIOINT to realize its potential. Since 2014, data sharing considerations have been governed by the principles of access and benefit sharing as laid out in the Nagoya Protocol.^29^ However, there is growing recognition that the operationalization of these principles must look very different in this era of large databases of digital sequence information, particularly as these data may be used to train large language models and the links between use of a genetic resource and commercial end products become more difficult to trace. As a special case, bioinformatic data on pathogens is far from a global commons, and tensions among historical global health norms, security and economic ramifications of sharing dangerous sequences, and assertions of pathogen data as a sovereign genetic resource have surfaced repeatedly over the past decade, starkly so during the COVID-19 pandemic.^30^

Data sharing infrastructure can smooth the exchange of bioinformation. For instance, the Global Initiative for Sharing All Influenza Data (GISAID) operates through a licensing mechanism that facilitates access while preserving data ownership, with major implications for facilitating global COVID-19 responses (despite recent difficulties with transparent implementation).^30^ The ongoing negotiations of World Health Organization member states on a potential pandemic accord also attempt to address underlying structural barriers inhibiting equitable access to the benefits of pathogen genomic data, including medical countermeasures,^31^ though these are largely limited in scope to traditional state actors and come with complex geopolitical legacies. Enhancing the global distribution of capabilities to generate and utilize pathogen data addresses these concerns, but on the other hand, the recent proliferation of efforts to build biosafety level 3 and 4 laboratories raises new concerns about amplifying biosafety, biosecurity, and dual-use risks.^32^

Another axis of data sharing requiring vigilance is individual privacy. Environmental samples, such as wastewater samples, may contain some amount of human DNA^33^ and have been used to track chemical substances, like drugs, in conjunction with law enforcement agencies. There are legitimate concerns about the lack of required informed consent for environmental sampling and the potential for misuse of any human genomic data to target certain populations, even as individuals are generally not identifiable from these samples. These topics require policy and regulatory attention, and BIOINT practitioners have a responsibility to work with communities to establish transparent and rigorous ethical standards for privacy protection and data usage.

The specific legacies of data sharing vary for clinical pathogen data versus environmental metagenomic data, but the overarching complexities apply to BIOINT writ large. Addressing them with creative approaches to knowledge exchange infrastructure is critical to achieving the promise of BIOINT. Much-needed incentives for multilateral sharing will rely on mechanisms for equitable access, benefit sharing, ownership and licensing, and ethical standards pertaining to BIOINT data.

### Operationalization

Timely action based on BIOINT is triggered by an anomalous event—a deviation from the environmental baseline, wherein a sequence shows up in an unusual place, with unusually high abundance, spreads or evolves in an unusual way, or is previously unseen and unknown. Advances in bioinformatic and epidemiological analysis are making it easier, faster, and more reliable to identify anomalies within vast troves of data. These insights, when validated and contextualized by additional layers of BIOINT, help leaders answer key questions about the event in order to respond. Sample metadata and integrated geospatial data on population density, readiness, and the proximity of critical infrastructure near the location of an anomaly helps rapidly triage events that are most likely to cause major harm and direct emergency response resources accordingly.

Another immediate need is to characterize transmission dynamics to deploy interventions that will dampen spread. At the front end, BIOINT helps elucidate pathways to emergence and identify points of proactive intervention.^34^ In most cases, once a pathogen has emerged, insights into human host mobility data are central. On a global scale, overlaying air travel patterns helps foresee the likely exportation of a threat to other countries. On a more localized scale, dispersal models informed by data on viral genomes along with geography, climate, and demography help understand and predict the magnitude and duration of transmission clusters for different types of threats, thus helping to design more effective interventions.^35^

Critically, the Intelligence Advanced Research Projects Activity (IARPA) recently announced a novel slate of technologies to determine whether a sample is engineered, which could be used to rapidly ascertain whether an anomalous threat appears to have been modified to, for instance, increase virulence or thwart countermeasures.^36^ This technology is expected to progress in coming years to be able to detect an expanded set of engineering signatures in increasingly complex environmental samples. Early warning signs of deliberate manipulation and potentially intentional release could trigger more rapid involvement of the security apparatus in mounting an appropriate response to minimize adverse consequences.

## Conclusion

To accelerate global health security amid a diverse and growing set of biological threats, BIOINT must be a core organizational paradigm for the biological century and a core component of the new wave of multilateral intelligence. Biotechnological advancements are giving us unprecedented access to information about the world's biological environment. Gathering and making sense of that information is a vital and urgent task as we seek to build a world free from the looming specter of the next global pandemic and resilient to the destabilizing effects of a crowded and manipulable information landscape. Moreover, as with satellite imagery, a wide variety of important collateral applications exist, ranging from environmental monitoring to enhancing bioengineering, to help unleash a sustainable bioeconomy. This is an ambitious project that certainly will not be built all at once—but the tools to realize this vision are within our grasp. We need a collective commitment to laying the global foundations to collect, analyze, and share biological intelligence.
